# Increased Expression of the Leptin Gene in Adipose Tissue of Patients with Chronic Kidney Disease–The Possible Role of an Abnormal Serum Fatty Acid Profile

**DOI:** 10.3390/metabo10030098

**Published:** 2020-03-08

**Authors:** Justyna Korczyńska, Aleksandra Czumaj, Michał Chmielewski, Maciej Śledziński, Adriana Mika, Tomasz Śledziński

**Affiliations:** 1 Department of Pharmaceutical Biochemistry, Faculty of Pharmacy, Medical University of Gdansk, 80-211 Gdansk, Poland; justyna.korczynska@gumed.edu.pl (J.K.); aleksandra.czumaj@gumed.edu.pl (A.C.); 2 Department of Nephrology, Transplantology and Internal Medicine, Faculty of Medicine, Medical University of Gdansk, 80-211 Gdansk, Poland; chmiel@gumed.edu.pl; 3 Department of General, Endocrine and Transplant Surgery, Faculty of Medicine, Medical University of Gdansk, 80-214 Gdansk, Poland; msledz@gumed.edu.pl; 4 Department of Environmental Analytics, Faculty of Chemistry, University of Gdansk, 80-309 Gdansk, Poland; adrianamika@tlen.pl

**Keywords:** chronic kidney disease, leptin, fatty acids, adipose tissue, adipocytes

## Abstract

Chronic kidney disease (CKD) is associated with an increased level of leptin and an abnormal fatty acid (FA) profile in the serum. However, there are no data on the associations between them, and the reason for increased serum levels in patients with CKD is not well elucidated. Recently, we found that a CKD-related abnormal FA profile caused significant changes in the expression of genes involved in lipid metabolism in hepatocytes. The aim of this study was to examine whether leptin gene expression in subcutaneous adipose tissue (SAT) of patients with CKD may contribute to increased serum levels of this adipokine and whether the abnormal serum FA profile observed in CKD patients has an impact on leptin gene expression in adipocytes. The FA profile was measured in serum samples from patients with CKD and controls by GC–MS. The relative mRNA levels of leptin were measured in SAT by Real-Time PCR. Moreover, the effect of the CKD-related abnormal FA profile on leptin gene expression was studied in in vitro cultured 3T3-L1 adipocytes. Patients with CKD had higher concentrations of serum leptin than controls and higher expression level of the leptin gene in SAT. They also had increased serum monounsaturated FAs and decreased polyunsaturated FAs. The incubation of adipocytes with FAs isolated from CKD patients resulted in an increase of the levels of leptin mRNA. Increased leptin gene expression in SAT may contribute to elevated concentrations of these adipokine in patients with CKD. CKD-related alterations of the FA profile may contribute to elevated serum leptin concentrations in patients with CKD by increasing the gene expression of this adipokine in SAT.

## 1. Introduction

Chronic kidney disease (CKD), with its prevalence exceeding 5% of the general population, constitutes an important clinical issue [1]. Irrespective of the underlying disease, CKD increases the cardiovascular burden of the patients several times compared to people with preserved kidney function [2]. Potential mechanisms for increased cardiovascular risk in CKD include alterations in the lipid profile and serum adipokine levels [3,4,5]. Changes in adipokine levels have been discussed in the context of CKD progression and the risk of comorbidities [6,7,8,9]. Leptin is one of the hormones secreted by adipose tissue and has been recognized as a signaling molecule that regulates energy homeostasis [10,11]. This protein also plays an important role in immune regulation and inflammation, both closely associated with oxidative stress and endothelial dysfunction, which may affect the risk of cardiovascular disorders [12,13,14]. Previous studies showed increased concentrations of leptin in the serum of patients with CKD [7,15]; however, the mechanism of this change is not well elucidated.

CKD is also associated with significant lipid disorders. The most frequently described changes focus on cholesterol and triacylglycerols. Many studies, including our previous research, have also shown abnormal serum fatty acid (FA) profiles in patients with CKD [16,17,18]. Dyslipidemia observed in patients with CKD is one of the well-known risk factors of cardiovascular disease (CVD) or diabetes mellitus [19,20,21]. This is especially important because cardiovascular disease is the leading cause of death in this group of patients [19]. Changes in the lipid profile in patients with CKD may also actively participate in the deterioration of renal function, thus contributing to the worsening of the disease [20,22]. However, all of the consequences of these alterations are still not fully understood.

Leptin and FAs are critical factors for the crosstalk between adipose tissue and other metabolically important organs, including the kidneys. Although elevated leptin levels and alterations in the fatty acid profile are factors that can be involved in the pathogenesis and complications of CKD, no previous study has investigated the relationship between them. It is still unclear whether changes in leptin levels in CKD are caused by a reduced glomerular filtration rate, increased production in adipose tissue, or both. Our previous study showed that an altered FA profile in patients with CKD significantly changed hepatocyte metabolism [16]. Thus, in the present study, we aimed to examine the associations of leptin serum levels and its gene expression in adipose tissue. Moreover, we studied the effect of an abnormal serum FA profile in patients with CKD on the expression of the leptin gene in adipocytes.

## 2. Results

### 2.1. Leptin Levels in Serum and mRNA Levels in Subcutaneous Adipose Tissue of Study Subjects

**Figure 1:**
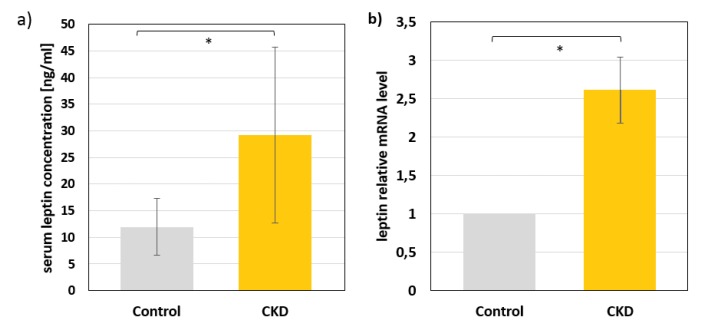
The serum concentrations of leptin (**a**) and leptin mRNA levels (**b**) in subcutaneous fat tissue of patients with chronic kidney disease (CKD) and control subjects. Data are shown as the mean ± SD, * *p* < 0.05. Serum leptin concentrations were assayed in 46 CKD patients and 57 healthy subjects. Leptin mRNA levels were assayed in adipose tissue obtained from 22 patients with CKD and 11 healthy subjects.

The mean serum leptin concentrations among patients with CKD (29.24 ± 16,5 ng/mL) were significantly elevated, at almost three times higher than the mean value for healthy controls (11.96 ± 5,3 ng/mL) (Figure 1a). When we analyzed serum concentrations of leptin separately in women and men, we found that both male and female CKD patients had almost three times higher serum leptin concentrations than controls; however, the leptin concentrations in women (both in CKD patients and controls) were about two times higher than in men (in CKD women 37.7 ± 15 vs. 15.8 ± 9.9 in healthy women, *p* < 0.01; in CKD men 19.6 ± 10 vs. 7.54 ± 5.2 in healthy man *p* < 0.05). The sex-related differences in serum leptin concentrations are in agreement with results of other researchers [23]. The relative mRNA level of the leptin gene in subcutaneous adipose tissue of patients with CKD was approximately three times higher than that in controls (Figure 1b). 

### 2.2. Serum Fatty Acid Profile of Study Subjects

The profile of FA in the serum of control subjects and patients with CKD is shown in Table 1. We observed several alterations in the FA profile, including various individual FAs and main groups of FAs (Table 1). The total saturated FA (SFA) and monounsaturated FA (MUFA) contents in the serum were significantly higher in the CKD group than those in the control group. At the same time, patients with CKD had lower levels of total n-3 polyunsaturated FAs (n-3 PUFAs) and n-6 polyunsaturated FAs (n-6 PUFAs) in the serum. This section may be divided by subheadings. It should provide a concise and precise description of the experimental results, their interpretation, and the experimental conclusions that can be drawn.

### 2.3. The Effect of the CKD-Related Abnormal Fatty Acid Profile on the Expression of Leptin in In Vitro Cultured Adipocytes

**Figure 2:**
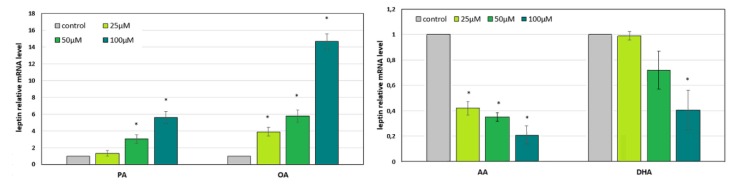
The relative mRNA level of leptin in 3T3-L1 adipocytes cultured for 48 h with various concentrations of palmitic acid 16:0 (PA), oleic acid 18:0 (OA), arachidonic acid 20:4 n-6 (AA), and docosahexaenoic acid 22:6 n-3 (DHA) or without FA supplementation (control). * Significantly different compared to the control (*p* < 0.05). Data are presented as the mean ± SD. All experiments were run in three independent attempts.

To examine whether reported alterations in the proportion of particular serum FA groups in CKD had an impact on adipose leptin gene expression, we treated 3T3-L1 adipocytes with a selected representative SFA (palmitic acid 16:0, PA), MUFA (oleic acid 18:1, OA), n-3 PUFA (docosahexaenoic acid 22:6 n-3, DHA), and n-6 PUFA (arachidonic acid 20:4 n-6, AA). All FAs were used at three different concentrations (25, 50, and 100 µM). After 48 h of incubation, PA and OA, FAs that are elevated in the serum of patients with CKD, increased the expression of the leptin gene, whereas DHA and AA–FAs, which are decreased in the serum of patients with CKD, decreased the expression of the leptin gene (Figure 2). Almost all these changes were statistically significant. All observed effects were dose-dependent.

**Figure 3:**
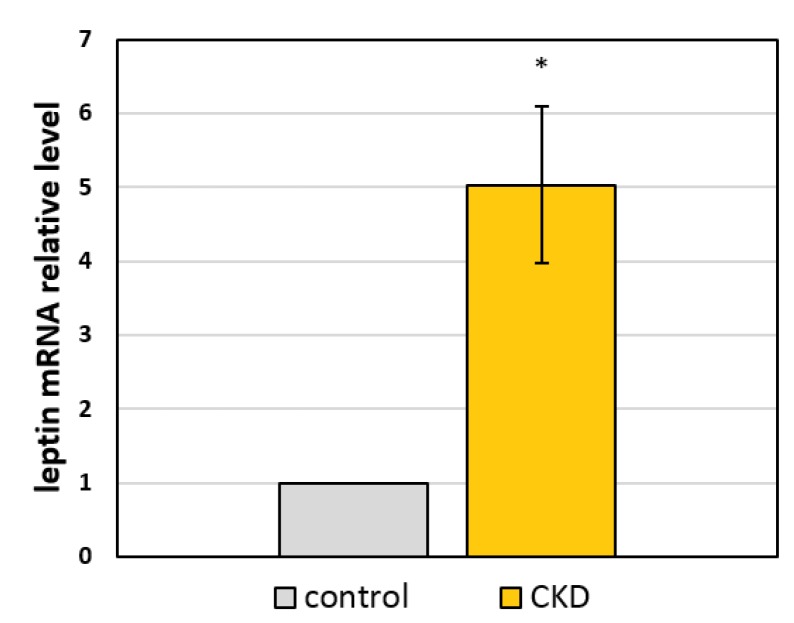
The relative mRNA level of leptin in 3T3-L1 adipocytes cultured for 48 h with a set of FAs extracted from the serum of control subjects (control) or patients with stage 5 CKD (CKD). * *p* < 0.05 compared to the control. Data are presented as the means ± SD. All experiments were run in three independent attempts.

**Figure 4:**
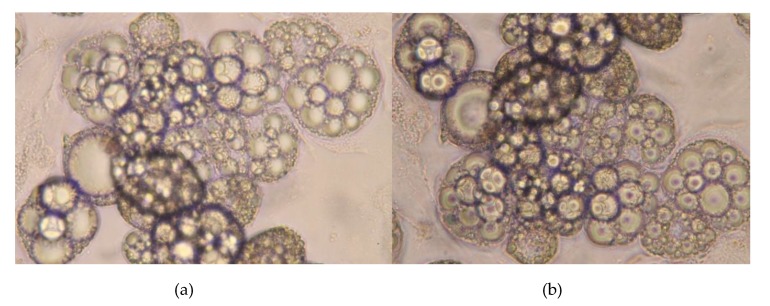
Representative sample of 3t3 adipocytes before (**a**) and after (**b**) treatment by FA isolated from serum of CKD patients.

We are aware that, in patients with CKD, alterations in the proportion of all particular serum FA groups occur at the same time. To examine the combined effect of all FA disorders, we decided to use a complete set of FAs isolated from CKD patients and healthy controls serum samples. Incubation of adipocytes with a set of FAs from patients with CKD resulted in a significantly increased mRNA level of the leptin gene in comparison to the mRNA level from adipocytes incubated with FA-mix from healthy subjects (Figure 3). We did not find any structural changes in the cells after treatment with a set of FAs isolated from patients with CKD or from healthy subjects (Figure 4).

## 3. Discussion

Our results demonstrated that, compared to healthy volunteers, patients with CKD had higher serum leptin levels. Concerning leptin, this result is in line with most clinical studies of leptin alteration in kidney diseases in adults and children [15,24,25,26,27]. Interestingly, a few studies have shown contradictory results [28,29,30]. However, in these cases, patients with CKD have experienced significant weight loss during dialysis. In our study, there was no difference in BMI between the CKD and control groups. In patients with CKD, an increased level of leptin may predict poor prognosis. A number of studies have indicated that leptin is involved in CKD progression and CKD complications [14,31]. Leptin plays a role in various types of kidney cells (e.g., glomerular mesangial cells, glomerular endothelial cells, and podocytes), and high leptin levels can lead to renal injury symptoms. Leptin inhibits the expression of nephrin, podocin, podoplanin, and podocalyxin (the podocyte-associated molecules necessary for the proper functioning of the renal filtration barrier) and promotes the production of reactive oxygen species (a known factor involved in the pathogenesis of CKD) [14,31]. The late stages of CKD are related to protein–energy wasting (reduced body protein and fat mass, and usually reduced protein and energy intake), and since leptin inhibits appetite, it may contribute to a further deterioration of nutritional status or even to malnutrition [32].

We show that increased serum levels of leptin observed in patients with CKD are accompanied by increased expression of the leptin gene in adipose tissue. Previous studies suggested that decreased kidney function (and as a consequence, decreased renal clearance of adipokines) may contribute to the elevated circulating levels of leptin in patients with CKD [33]. However, our study showed increased SAT mRNA levels and serum protein levels of leptin, which suggests that its production in SAT may contribute to its serum concentrations. To date, the literature data on this subject are very poor. Nordfors et al. reported elevated leptin gene expression only in adipose tissue in patients with chronic renal failure with inflammation compared to patients with chronic renal failure with no inflammation and no changes in leptin expression between patients with chronic renal failure and healthy controls [33]. Nonetheless, they examined a small number of patients (15 patients with chronic renal failure, including two with inflammation) and used in situ hybridization histochemistry for gene expression quantification. In turn, Witasp et al. reported downregulation of the leptin gene in abdominal subcutaneous adipose tissue of patients with advanced CKD with ‘uremic–metabolic syndrome’ [34]. Since there are no data on how the expression of the leptin gene is regulated in the adipose tissue of patients with CKD, we tried to find a molecular mechanism for the increased expression of leptin in the SAT of our patients.

The consequences of changes in the FA profile are usually considered in the context of dietary intake. Our recent study showed that CKD-related alterations of the FA profile influence hepatocyte metabolism. Thus, in the present study, we examined the hypothesis that CKD-related FA alteration can affect leptin gene expression in adipocytes. We found specific effects of FAs from various groups. However, since each FA can have a different effect on adipose tissue, alterations in the FA profile during CKD should be considered together as a whole. Thus, we made an effort to address this issue by using total FAs extracted from patients and control serum. To the authors’ knowledge, this is a unique research approach. Our data demonstrated, for the first time, that the altered serum FA profile observed in patients with CKD increased adipocyte expression of leptin. Thus, it may also be responsible for the increased expression of this adipokine in SAT and its elevated circulating levels.

The study has limitations that ought to be mentioned. Our data are derived from patients at one point of chronic progressive disease. Further studies should consider the cross-sectional selection of patients. Another limitation is that our study cohort was not very numerous and there are relatively large deviations in some parameters; however, the differences were statistically significant. Thus, our study can serve as a proof-of-concept report. The expression of leptin gene has been assayed only at the mRNA levels. In our in vitro experiment, we used only one cell line as an adipocyte model. Adipose tissue is a highly heterogeneous organ with cell- and depot-specific functions [35,36,37]. However, we chose 3T3-L1 differentiated adipocytes because they are a well-characterized and widely used cell line in metabolic, molecular, and endocrine studies, including studies on leptin expression levels [38,39,40,41].

In conclusion, our study showed that increased leptin gene expression in SAT may contribute to elevated concentrations of this adipokine in patients with CKD. Moreover, alteration of the serum FA profile observed in the course of CKD might contribute to CKD-related elevated serum leptin levels, through induction of its gene expression in adipocytes.

## 4. Materials and Methods

### 4.1. Study Subjects

Forty-six patients with stage 5 CKD (27 males and 19 females; pre-dialysis and dialyzed, recruited from the Outpatient Unit of the Department of Nephrology, Transplantology and Internal Medicine in the Medical University of Gdansk) and 57 healthy subjects or metabolically healthy subjects (31 males and 26 females) who underwent hernia surgeries, matched for age and weight, with no known kidney disease, were included in this study. Among the abovementioned participants, subcutaneous adipose tissue, which is available during kidney transplantation, was taken from 22 patients with CKD and from 11 metabolically healthy subjects who underwent hernia surgeries at the Department of Surgery, Medical University of Gdansk. After overnight fasting, blood samples were taken from all the participants of the study. The study was performed in agreement with the principles of the Declaration of Helsinki of the World Medical Association. Experimental protocols received approval from the Local Bioethics Committee at the Medical University of Gdansk (protocol numbers: NKBBN/664/2013-2014, NKBBN/614-276/2014). The general characteristics of the study subjects and selected laboratory parameters are presented in Table 2. Patients with CKD had higher creatinine, blood urea nitrogen, and triacylglycerols, in comparison with control individuals.

### 4.2. Materials and Reagents

From Avantor Performance Materials Poland (Gliwice, Poland) methanol, chloroform, dichloromethane, n-hexane (all HPLC-grade), hydrochloric acid, and potassium hydroxide were acquired. DMEM, glucose, bovine calf serum, glutamine, penicillin/streptomycin solution, fetal bovine serum, dexamethasone, 3-isobutyl-1-methylxanthine, insulin, Oil Red O solution, palmitic acid, oleic acid, docosahexaenoic acid, arachidonic acid phosphate-buffered saline, FAME mix, Nuclease-Free Water, boron trifluoride–methanol solution, and 19-methyleicosanoic acid were obtained from Sigma-Aldrich (St. Louis, MO, USA). Eppendorf laboratory consumables were used for experiments (Hamburg, Germany).

### 4.3. Serum Leptin Assay

For the detection of serum leptin concentrations, the Leptin Human ELISA Clinical Range kit (BioVendor, Brno, Czech Republic) was used according to the manufacturer’s instructions. In brief, samples from the control group and samples from the CKD group were incubated in microplate wells coated with polyclonal anti-human leptin antibodies. Bound leptin was detected by horseradish peroxidase-conjugated polyclonal anti-human leptin antibody. Tetramethylbenzidine was used as a substrate for peroxidase, and color intensity was determined by measuring the absorbance at 450 nm.

### 4.4. Serum Fatty Acid Profile Analysis

Total lipids were extracted from the serum of patients with CKD and healthy subjects, using the method described by Folch et al., with a mixture of chloroform:methanol (2:1, *v/v*) [42]. Then, the lipid extracts were dried by evaporation under a stream of nitrogen and alkaline hydrolyzed with 0.5 M KOH in methanol, at 90 °C, for 3 h. Next, the mixture was acidified with 6 M HCl, and 1 mL of water was added. Fatty acids were extracted three times, with 1 mL of n-hexane, and evaporated under a stream of nitrogen. To obtain fatty acid methyl esters (FAMEs), 10% boron trifluoride–methanol solution was added to each sample, which was then heated at 55 °C, for 90 min. After 1.5 h, 1 mL of water was added to the mixture, and FAMEs were extracted three times with 1 mL of n-hexane and dried under nitrogen stream. Fatty acid profiles were analyzed by gas chromatography–mass spectrometry (GC–MS), using a QP-2010SE apparatus (Shimadzu, Kyoto, Japan), as described previously [16]. In brief, a 30 m 0.25 mm i.d. ZB-5MSi capillary column was used (film thickness 0.25 μm). Temperature of the column was set between 60 and 300 °C (4 °C/min). Helium was used as a carrier gas at the column head pressure of 100 kPa, and FAME ionization was carried out with 70 eV electron energy. Full-scan mode was applied, with mass scan range *m/z* 45–700. Then, 19-methyleicosanoic acid was used as an internal standard. FAMEs were identified by comparison with reference standards (37 FAME Mix, Sigma-Aldrich, St. Louis, MO, USA) and NIST2011 reference library.

### 4.5. Adipocyte Culture, Differentiation, and Treatment

The 3T3-L1 cell line was obtained from American Type Culture Collection (Manassas, VA, USA). Pre-adipocytes were cultured in expansion medium (Dulbecco’s modified Eagle’s medium (DMEM) with 4.5 mg/mL of glucose supplemented with 10% bovine calf serum, 4 mM glutamine, 100 IU/mL of penicillin, and 100 IU/mL of streptomycin), at 37 °C, in a 5% CO2 incubator. Cells were seeded at approximately 3000 cells per cm^2^. Two days after reaching confluence, cells were differentiated by replacing the expansion medium with high-glucose DMEM containing 10% fetal bovine serum (FBS), 1.0 µM dexamethasone, 0.5 mM 3-isobutyl-1-methylxanthine (IBMX), and 1.0 µg/mL of insulin. After 48 h, the differentiation medium was replaced by DMEM with 10% FBS, 10 μg/mL of insulin, 4 mM glutamine, 100 IU/mL of penicillin, and 100 IU/mL of streptomycin. Media were replaced every other day. After 10 days, the adipocytes were fully differentiated (confirmed by Oil red O staining). The 3T3-L1 fully differentiated adipocytes were used for experiments. Cells were supplemented for 48 h, with one selected fatty acid: PA, OA, DHA, and AA or with the full FA set isolated from pooled serum of 10 randomly selected patients with stage 5 CKD, or 10 healthy controls. From pooled patient or control serum samples, total lipids were extracted and hydrolyzed, as previously described in Czumaj et al. (2019) [16]. A mixture of fatty acids isolated from the patients and controls sera was added to the cell culture at exactly the same concentration as in the study participants’ sera. The FA set isolated from the serum of patients with CKD was characterized by a higher content of MUFAs and SFAs and a lower content of n-3 and n-6 PUFAs than FAs isolated from serum of control subjects [16].

### 4.6. Gene Expression Analyses

Total RNA isolation from in vitro cultured adipocytes and adipose tissue depots was carried out, using an RNeasy Lipid Tissue Mini Kit (Qiagen, Hilden, Germany) according to the manufacturer’s instructions. The RNA concentration and integrity were assessed by an Experion automated electrophoresis system (Bio-Rad, Hercules, CA, USA). One microgram of RNA was reverse transcribed, using the RevertAid First Strand cDNA Synthesis Kit (Thermo Fisher Scientific, Waltham, MA, USA). Quantitative real-time PCR was carried out in a CFX Connect Real-Time System (Bio-Rad), using the SensiFAST SYBR No-ROX Kit (Bioline Meridian Bioscience, Cincinnati, OH, USA). The comparative Ct method (ΔΔCt) was used for relative quantification of gene expression. The β-actin gene was used for normalization.

### 4.7. Statistics

Data are presented as the mean ± SD. Statistical analyses were performed by using two-tailed the Student’s *t*-test for two-group comparisons (including biochemical and anthropometric characteristics of the study subjects presented in Table 2) or analysis of variance (ANOVA), followed by post hoc correction (Bonferroni) for multi-group comparisons. The threshold of statistical significance was defined as *p* < 0.05. During all analyses, every sample was run in duplicate. The cell culture experiment was run in three independent attempts. All statistical analyses were performed by using STATISTICA 12 (TIBCO Software Inc., Palo Alto, CA, USA).

## Figures and Tables

**Table 1:** The percent content of the main classes of fatty acids in the serum of patients with chronic kidney disease (CKD) and the control group. The data are presented as fatty acid proportions (%). Values are the mean ± SD.

FA	CONTROL	CKD
14:0	1.16 ± 0.31	1.11 ± 0.50
16:0	22.9 ± 1.62	24.1 ± 2.10 *
18:0	6.96 ± 0.72	6.80 ± 0.98
OTHER SFAs	1.22 ± 0.09	1.36 ± 0.09
TOTAL SFAs	32.3 ± 1.83	33.4 ± 3.02 *
14:1	0.07 ± 0.02	0.05 ± 0.03
16:1	2.81 ± 0.85	2.95 ± 0.79
18:1	25.7 ± 3.15	28.9 ± 3.63 *
OTHER MUFAs	0.49 ± 0.08	0.65 ± 0.11 *
TOTAL MUFAs	29.1 ± 1.08	32.6 ± 1.22 *
18:3 n-3	0.31 ± 0.11	0.20 ± 0.09 *
20:5 n-3	0.94 ± 0.60	0.61 ± 0.26 *
22:6 n-3	1.03 ± 0.43	0.83 ± 0.38 *
OTHER N-3 PUFAs	0.37 ± 0.1	0.34 ± 0.13
TOTAL N-3 PUFAs	2.66 ± 1.04	1.98 ± 0.71 *
18:2 n-6	26.1 ± 3.59	22.9 ± 4.79 *
20:4 n-6	5.31 ± 1.14	4.53 ± 1.31 *
OTHER N-6 PUFAs	1.42 ± 0.34	1.10 ± 0.32
TOTAL N-6 PUFAs	32.8 ± 3.82	28.6 ± 5.39 *

* Statistically significant compared to controls at *p* < 0.05. SFAs—saturated fatty acids; MUFAs— monounsaturated fatty acids; PUFAs—polyunsaturated fatty acids.

**Table 2:** Selected biochemical and anthropometric characteristics of the study subjects.

Parameter	CONTROL	CKD
AGE (years)	47 ± 14.9	51 ± 13.0
BMI (kg/m^2^)	26.0 ± 3.8	25.9 ± 4.8
CREATININE (mg/dL)	0.9 ± 0.2	6.15 ± 2.5 *
BUN (mg/dL)	15.1 ± 3.6	44.8 ± 25.0 *
ALBUMIN (g/L)	39.5 ± 3.9	37.5 ± 4.6
CRP (mg/dL)	2.1 ± 2.5	4.9 ± 6.1 *
TG (mg/dL)	115.6 ± 59.6	150.2 ± 73.7 *
TC (mg/dL)	195.9 ± 45.5	200.6 ± 48.0
GLUCOSE (mg/dL)	96.2 ± 20.2	102.7 ± 27.8
INSULIN (mU/mL)	9.4 ± 5.4	9.6 ± 6.2
HOMA-IR	2.35 ± 2.0	2.8 ± 2.7

* Statistically significant compared to controls at *p* < 0.05. Values are the mean ± SD. BMI—body mass index; BUN—blood urea nitrogen; CRP—C-reactive protein; HOMA-IR—homeostatic model assessment of insulin resistance; TG—triacylglycerols TC—total cholesterol.

## Abbreviations

AA	Arachidonic acid
BMI	Body mass index
BUN	Blood urea nitrogen
CKD	Chronic kidney disease
CRP	C-reactive protein
CVD	Cardiovascular disease
DHA	Docosahexaenoic acid
DMEM	Dulbecco‘s modified Eagle‘s medium
FA	Fatty acid
FAME	Fatty acid methyl esters
FBS	Fetal bovine serum
GC-MS	Gas chromatography-mass spectrometry
HOMA-IR	Homeostatic model assessment of insulin resistance
IBMX	3-isobutyl-1-methylxanthine
MUFA	Monounsaturated fatty acid
OA	Oleic acid
PA	Palmitic acid
PUFA	Polyunsaturated fatty acid
SAT	Subcutaneous adipose tissue
SFA	Saturated fatty acid
TC	Total cholesterol
TG	Triacylglycerols
